# Perceived burdensomeness and suicidal ideation: parallel mediating roles of non-suicidal self-injury and digital self-harm and the moderating role of psychological capital

**DOI:** 10.3389/fpsyg.2026.1818894

**Published:** 2026-05-04

**Authors:** Xuefei Li, Chengting Ju, Yuxi Guo

**Affiliations:** Psychological Counseling Centre, Chang’an University, Xi’an, China

**Keywords:** digital self-harm, non-suicidal self-injury, perceived burdensomeness, psychological capital, suicidal ideation

## Abstract

**Background:**

Suicidal ideation is a key predictor of suicidal behavior, yet less is known about how interpersonal risk factors translate into suicidal ideation through both offline and online self-harm behaviors, and under what conditions these pathways may be buffered. Grounded in the Interpersonal-Psychological Theory of Suicide, this study examined the parallel mediating roles of non-suicidal self-injury (NSSI) and digital self-harm in the association between perceived burdensomeness and suicidal ideation, as well as the moderating role of psychological capital.

**Methods:**

Using a cross-sectional design, data were collected from 2,167 Chinese college students (68.6% female; *M_age_* = 18.91 years, *SD* = 1.31) via an online survey.

**Results:**

Parallel mediation analyses showed that perceived burdensomeness was significantly associated with suicidal ideation both directly and indirectly through NSSI and digital self-harm. Moderated mediation analyses further revealed that psychological capital significantly moderated the association between digital self-harm and suicidal ideation, but not the association between NSSI and suicidal ideation. Specifically, digital self-harm was positively related to suicidal ideation only among individuals with low psychological capital.

**Conclusion:**

These findings highlight the dual behavioral pathways linking perceived burdensomeness to suicidal ideation and identify psychological capital as a context-specific protective factor that buffers suicide risk in digital self-harm contexts.

## 1 Introduction

Suicide has become a major global public health challenge. The World Health Organization (2021) reports that suicide is the third leading cause of death among individuals aged 15–29. In China, adolescent suicide is also a serious concern, with suicide mortality rates approximately 1.5 times higher than the global average (Glenn et al., 2020). Suicidal ideation refers to thoughts about engaging in suicidal behavior or ending one’s life (Hatkevich et al., 2019) and is considered a core indicator of suicide risk. Evidence suggests that about 20% of individuals who experience suicidal ideation eventually attempt suicide (Borges et al., 2010). Therefore, systematically identifying the risk pathways and protective mechanisms underlying suicidal ideation is essential for advancing suicide theories and developing effective prevention strategies.

### 1.1 Perceived burdensomeness and suicidal ideation

The Interpersonal-Psychological Theory of Suicide (IPTS) provides a significant framework for understanding the emergence of suicidal ideation (Joiner, 2005). This theory posits that thwarted belongingness and perceived burdensomeness are two proximal interpersonal risk factors for suicidal ideation. Perceived burdensomeness refers to the belief that one is a burden to others and that one’s death is more valuable than one’s life (Van Orden et al., 2010; Assavedo and Anestis, 2015). Extensive evidence indicates that, compared with thwarted belongingness, perceived burdensomeness shows a more stable and stronger association with suicidal ideation (Chu et al., 2017; Ma et al., 2016). A meta-analysis by Chu et al. (2017), which synthesized over 100 studies, identified perceived burdensomeness as one of the most robustly supported pathways in IPTS. Importantly, perceived burdensomeness independently predicted suicidal ideation even after controlling for depression, hopelessness, and functional impairment. Subsequent studies have replicated this finding across diverse cultures and populations (Cukrowicz et al., 2011; Shim et al., 2021), underscoring its role as a core indicator of suicide risk.

Although strong evidence confirms perceived burdensomeness as one of the most powerful predictors of suicidal ideation, most studies have focused on its direct linear association with suicidal ideation. Far less attention has been paid to the internal mechanisms through which perceived burdensomeness may translate into more lethal suicidal ideation via maladaptive behaviors. In the digital era, interpersonal distress often manifests in both offline behaviors (e.g., non-suicidal self-injury) and online behaviors (e.g., digital self-harm). However, few studies have examined these two forms of self-harm simultaneously within the IPTS framework. Moreover, prior research has primarily emphasized “risk amplification” processes, while largely neglecting the “buffering” role of positive psychological resources. Psychological capital, as a core internal resource against psychopathology, may mitigate the effects of perceived burdensomeness on self-harm and suicidal ideation, yet systematic evidence remains limited. Accordingly, this study adopts a behavioral mechanism perspective and proposes a moderated mediation model to examine the parallel mediating roles of non-suicidal self-injury and digital self-harm in the association between perceived burdensomeness and suicidal ideation, as well as the protective moderating effect of psychological capital.

### 1.2 The mediating role of NSSI and digital self-harm

A growing body of research indicates that suicidal ideation is often not directly triggered by interpersonal risk factors, but rather develops progressively through a series of maladaptive behaviors. Among these, non-suicidal self-injury (NSSI) is considered a key behavioral pathway linking interpersonal distress to suicide risk (Chu et al., 2017). The Interpersonal Theory of Suicide (IPTS) posits that chronic experiences of perceived burdensomeness and thwarted belongingness intensify emotional distress and self-devaluation, which may lead individuals to engage in NSSI as a means of emotion regulation, self-punishment, or interpersonal communication (Joiner, 2005; Nock, 2010). According to the four-function model of self-injury (Nock, 2010; Klonsky, 2007), NSSI can alleviate distress through negative intrinsic reinforcement while also securing attention and support via positive interpersonal reinforcement, thereby temporarily “repairing” unmet interpersonal needs. Substantial empirical research supports the close association between perceived burdensomeness and NSSI (Chen et al., 2024; Shen et al., 2024a, b). Moreover, repetitive self-injury not only reflects escalating psychological pain but may also foster acquired capability for suicide by reducing fear of death and increasing pain tolerance, ultimately strengthening suicidal ideation (Assavedo and Anestis, 2015; Chu et al., 2017). Thus, NSSI is widely regarded as a critical mediator linking interpersonal risk factors to suicidal ideation.

Notably, as social interactions among adolescents and young adults increasingly migrate to cyberspace, self-harm behaviors have extended into online contexts. Digital self-harm refers to the anonymous posting, sending, or dissemination of self-deprecating, harmful, or threatening content about oneself on online platforms (Patchin and Hinduja, 2017). Existing evidence indicates that individuals who engage in digital self-harm behaviors have a significantly increased risk of developing suicidal thoughts and attempting suicide (Patchin et al., 2023; Wang et al., 2024). While digital self-harm and traditional NSSI share overlapping functions, such as emotion regulation and interpersonal signaling (Patchin and Hinduja, 2024), they are distinct in their socio-technical contexts. Compared to private and physical NSSI, digital self-harm embeds self-injury within a public, evaluable, and persistent socio-technical environment, potentially amplifying individuals’ feelings of worthlessness through immediate interpersonal feedback loops. These functional similarities and contextual distinctions suggest that NSSI and digital self-harm represent distinct yet parallel modalities (offline vs. online) of self-harm. Nevertheless, existing research rarely examines the parallel mediating effects of both NSSI and digital self-harm on perceived burden and suicidal ideation within the same theoretical framework. Consequently, it is necessary to integrate offline and online self-harm behaviors into a unified model to systematically explore their unique and shared pathways in the process from perceived burden to suicidal ideation.

### 1.3 The moderating role of psychological capital

Although perceived burden and self-harm behaviors significantly increase suicide risk, the progression from “risk exposure” to “suicidal ideation” is not inevitable, suggesting the moderating influence of protective factors. Psychological capital as a higher-order positive psychological resource encompassing core components such as hope, optimism, self-efficacy, and resilience, is regarded as a crucial internal buffering system for individuals to cope with adversity and maintain psychological functional equilibrium (Luthans et al., 2007a, b). From a positive psychology perspective, individuals with high psychological capital typically exhibit greater coping flexibility and emotional resilience. They effectively counteract the destructive impact of negative events through future-oriented positive beliefs (hope) and confidence in their coping abilities (self-efficacy) (Abbas and Raja, 2015; Avey et al., 2011).

In the field of suicide research, a growing body of evidence indicates a stable negative association between psychological capital and suicidal ideation. Empirical findings indicate that psychological capital directly predicts lower levels of suicidal ideation and indirectly reduces suicide risk by alleviating depression, hopelessness, and emotional dysregulation (Zheng et al., 2025; Song and Song, 2024; Zhang et al., 2025). Based on this evidence, psychological capital likely serves as a critical “buffer” in the dynamic pathway of suicide risk. Specifically, while perceived burden may increase suicidal ideation risk through NSSI and digital self-harm, individuals with higher psychological capital are more likely to mobilize adaptive coping resources even when engaging in self-injury, thereby reducing the likelihood of transition from self-injury to suicidal ideation (Liu et al., 2023). However, existing research lacks examination of whether psychological capital can simultaneously mitigate the effects of offline self-injury and digital self-harm on suicidal ideation. Therefore, this study incorporates psychological capital into the model to test its moderating role in the relationship between self-injury behaviors and suicidal ideation, thereby clarifying its protective function within critical risk pathways.

### 1.4 The current study

Grounded in the Interpersonal-Psychological Theory of Suicide and functional models of self-harm, this study aims to examine how perceived burdensomeness influences adolescents’ suicidal ideation. We test the parallel mediating roles of NSSI and digital self-harm, as well as the moderating role of psychological capital. We propose three hypotheses:

1. Perceived burdensomeness positively predicts suicidal ideation among Chinese college students.
2. NSSI and digital self-harm play parallel mediating roles in the association between perceived burdensomeness and suicidal ideation.
3. Psychological capital moderates the associations between both forms of self-harm and suicidal ideation, with a more pronounced buffering effect expected for the digital self-harm pathway among adolescents with lower psychological capital.

**Figure 1:**
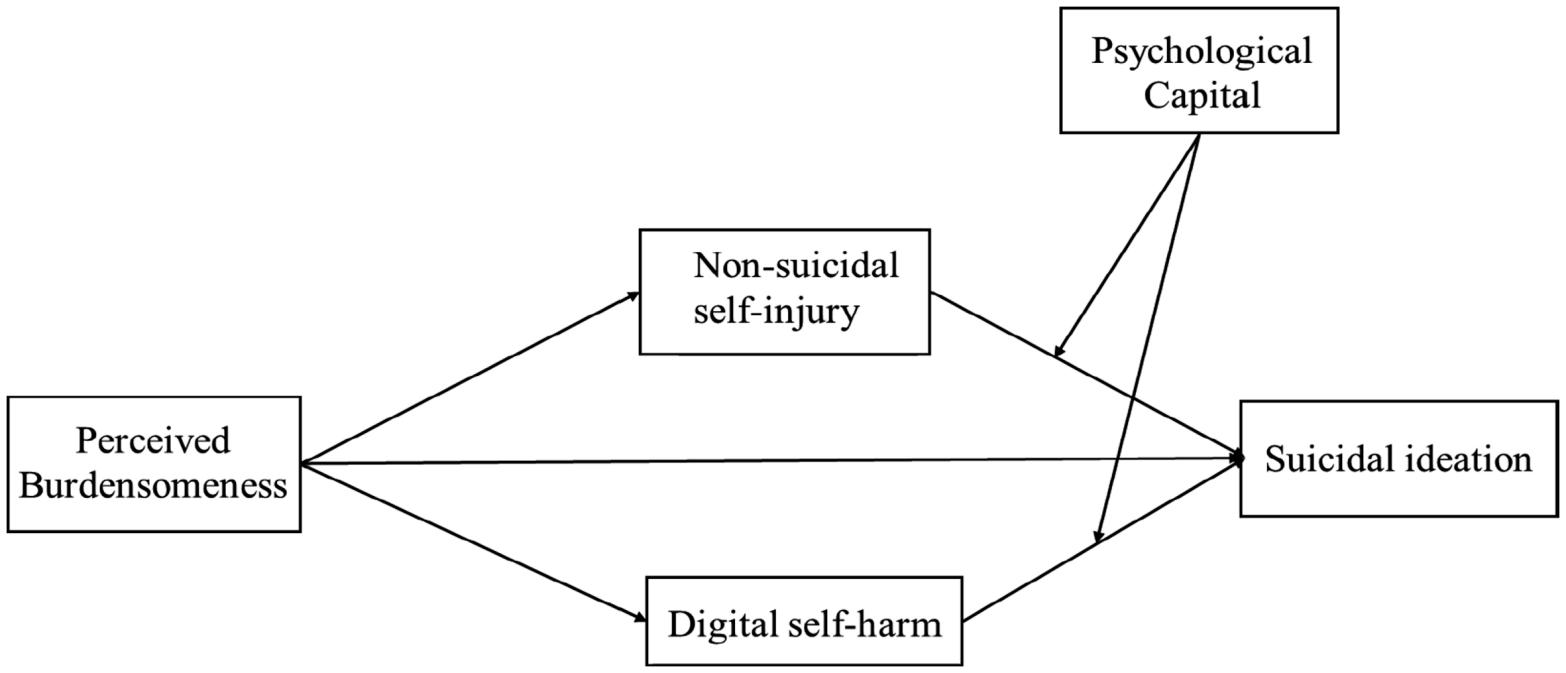
Hypothesized moderated mediation model from perceived burdensomeness to suicidal ideation.

The proposed moderated mediation model is presented in Figure 1.

## 2 Methods

### 2.1 Participants

A total of 2,719 first-year students from a university in Guangdong Province, China, completed the online survey. Several exclusion criteria were applied to enhance data quality: (a) abnormally short or long completion times (i.e., response times exceeding three standard deviations from the mean), (b) incorrect identity information, and (c) invalid response patterns (e.g., inconsistent answers to similar items). This resulted in a final sample of 2,167 valid responses. Among the final participants, 68.6% were female. The average age of participants was 18.91 years (*SD* = 1.31). To examine whether our exclusion criteria introduced selection bias, we compared the final sample (*N* = 2,167) with excluded participants (*N* = 552) on demographic variables. Independent-samples t-test revealed no significant age difference between groups (*p* > 0.05), and a chi-square test indicated no significant gender difference (*p* > 0.05). These results suggest that our exclusion criteria did not introduce systematic bias.

### 2.2 Measures

#### 2.2.1 Perceived burdensomeness

This study used the Perceived Burdensomeness subscale of the Chinese Revised Interpersonal Needs Questionnaire (INQ; Li et al., 2015). This subscale includes 6 items, each rated on a 7-point scale ranging from 1 (“completely disagree”) to 7 (“completely agree”), with higher scores indicating greater perceived burdensomeness. This subscale has demonstrated good reliability among Chinese adolescents (Chen et al., 2024). In the present study, the Cronbach’s *α* coefficients for this subscale is 0.848.

#### 2.2.2 Digital self-harm

Youth engagement in digital self-harm was measured using a single-item indicator adapted from Meldrum et al. (2022). Participants were asked: “During the past 30 days, have you posted or shared disparaging information about yourself anonymously online or on social media for the purpose of self-harm?.” Responses were recorded on a 5-point scale ranging from 0 (*no*) to 4 (*10 or more times*). This single-item approach has been increasingly utilized in recent empirical studies to capture the prevalence of digital self-harm (e.g., Zhao et al., 2026; Zeng et al., 2026).

#### 2.2.3 Non-suicidal self-injury (NSSI)

NSSI was measured using 12 items adapted from the Deliberate Self-Harm Inventory (DSHI; Gratz, 2001). Participants reported whether they had intentionally engaged in the listed behaviors without suicidal intent during the past 6 months. Each item was rated on a 6-point scale from *0 = never* to 5 *= five times or more*, with higher scores reflecting greater frequency of NSSI. This measure has demonstrated good reliability and validity among Chinese adolescents (Shen et al., 2024a, b). In the current sample, Cronbach’s *α* was 0.873.

#### 2.2.4 Psychological capital

Participants’ psychological capital was measured using the Chinese PCQ (Luthans et al., 2007a, b; Wen et al., 2009). Each item was rated on a 6-point Likert scale (1 = very inconsistent, 6 = very consistent), where higher values represent greater psychological capital over the past six months. The scale has been proved to have good reliability among Chinese college students (Xiang and Jiang, 2025). In this study, the Cronbach’s α coefficient of this scale was 0.953.

#### 2.2.5 Suicidal ideation

Suicidal ideation was measured using the sixth item of the BSI-CV: *“During the past six months, how many times have you seriously thought about killing yourself?”* Responses were rated on a 6-point scale from 0 (“never”) to 5 (“five times or more”), and any score above 0 was considered indicative of suicidal ideation. This specific item has been used as a standalone indicator of suicidal thoughts in previous research (e.g., Lemke et al., 2025).

### 2.3 Procedures

The survey was administered through Wenjuanxing, a professional online questionnaire platform. After accessing the digital link, participants were briefed on the study’s scope and assured that their data would be handled with strict confidentiality, accessible exclusively to the research team. Ethical clearance was granted by the Institutional Review Board (IRB) of the principal investigator’s university. Prior to data collection, formal informed consent was obtained from both the school administration and the participating students.

### 2.4 Data analysis

First, descriptive statistics and correlations among the study variables were computed using SPSS 24.0. Next, a parallel mediation model was tested to examine the relationship between perceived burdensomeness and suicidal ideation, with NSSI and digital self-harm as mediators. Gender and age were included as covariates in the model. The analysis was conducted using the PROCESS macro (model 4; Hayes, 2022). To obtain standardized results, all variables were standardized before running the multiple mediation analysis. The bootstrap method with 5,000 samples was used to computed the upper and lower limits of 95% confidential intervals and their standard errors (boot SE) for indirect effects. A significant indirect effect was indicated when zero was not included in the confidential intervals. Finally, a moderated parallel mediation model was tested to explore the moderating role of psychological capital using the PROCESS macro (model 14).

## 3 Results

### 3.1 Descriptive statistics

Table 1 presents the means, standard deviations, and correlations for the measured variables. Perceived burdensomeness, digital self-harm, NSSI, and suicidal ideation were significantly and positively correlated with each other. Psychological capital was significantly and negatively correlated with the other four variables.

**Table 1:** Bivariate correlations between and descriptive statistics of study variables (*N* = 2,167).

Variable	1	2	3	4	5	6	7
1. Perceived burdensomeness	—						
2. NSSI	0.27**	—					
3. Digital self-harm	0.32**	0.28**	—				
4. Suicidal ideation	0.40**	0.42**	0.42**	—			
5. Psychological capital	−0.41**	−0.18**	−0.32**	−0.40**	—		
6. Gender	−0.01	0.02	0.08**	0.15**	−0.23**	—	
7. Age	0.07**	0.09**	0.02	0.03	0.05*	−0.03	—
*Mean*	13.84	0.86	0.30	0.47	61.62	—	18.91
*SD*	8.27	3.73	0.78	1.26	15.45	—	1.31

*p < 0.05; **p < 0.01; NSSI = non-suicidal self- injury.

### 3.2 Testing of the parallel mediation model

The parallel mediation analysis indicated that perceived burdensomeness significantly predicted both NSSI (*β* = 0.32, *p* < 0.001) and digital self-harm (*β* = 0.32, *p* < 0.001), controlling for age and gender. Both NSSI (*β* = 0.32, *p* < 0.001) and digital self-harm (*β* = 0.22, *p* < 0.001) were positively associated with suicidal ideation, and perceived burdensomeness also had a significant direct effect on suicidal ideation (β = 0.22, *p* < 0.001).

The total effect of perceived burdensomeness on suicidal ideation was significant (estimate = 0.39, 95% CI [0.35, 0.43]), as was the total indirect effect through NSSI and digital self-harm (estimate = 0.17, 95% CI [0.14, 0.21]). Specifically, the indirect effect via NSSI was 0.10 (95% CI [0.08, 0.13]) and via digital self-harm was 0.07 (95% CI [0.05, 0.09]), indicating that both mediators partially explained the relationship between perceived burdensomeness and suicidal ideation. The parallel mediation model explained 34.7% of the variance in suicidal ideation (*R^2^* = 0.35, *F* (3, 2,163) = 382.71, *p* < 0.001), corresponding to a large effect size (Cohen’s f^2^ = 0.53).

### 3.3 Testing of the moderated parallel mediation model

**Figure 2:**
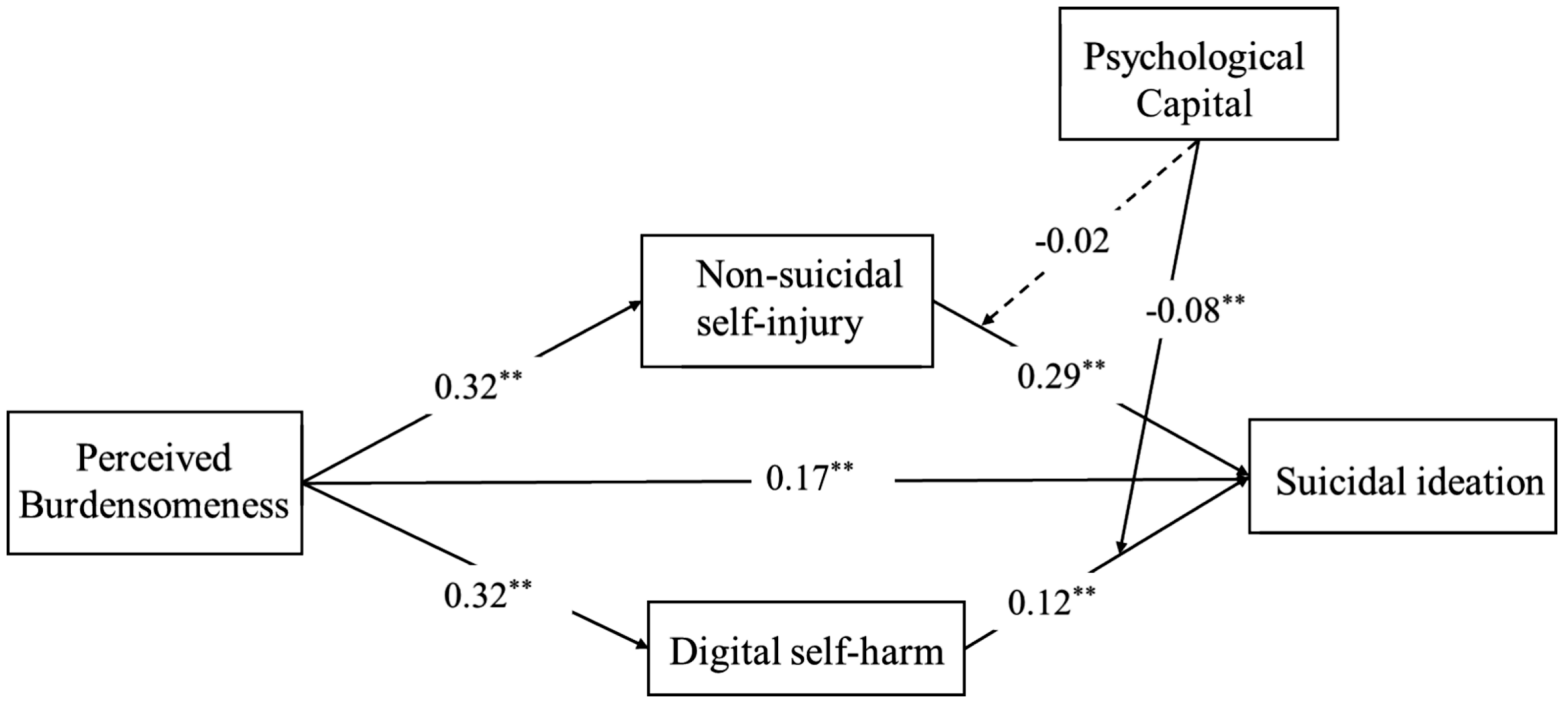
Standardized path coefficients for the moderated mediation model of perceived burdensomeness, non-suicidal self-injury, digital self-harm, psychological capital and suicidal ideation. Dotted lines indicate non-significant paths. For clarity, paths from demographic control variables (gender and age) are not displayed. *p < 0.05; **p < 0 0.01.

After establishing the mediation model, we examined the hypothesized moderated mediation model as described in Figure 1. Standardized path parameters for the model are presented in Table 2 and Figure 2. The overall model explained 40.1% of the variance in suicidal ideation (*R^2^* = 0.40, *F* (8, 2,139) = 179.01, *p* < 0.001), corresponding to a large effect size (Cohen’s f^2^ = 0.67). Psychological capital significantly moderated the relationship between digital self-harm and suicidal ideation (*β* = −0.08, *p* < 0.001), but the interaction between NSSI and psychological capital on suicidal ideation was not significant (*β* = −0.03, *p* = 0.081). Thus, the moderating effect of psychological capital emerged only for digital self-harm, not for NSSI.

**Table 2:** Standardized results of moderated parallel mediation model (*N* = 2,167).

Predictor variable	Model 1 NSSI		Model 2 Digital self-harm		Model 3 Suicidal ideation	
	β	t	β	t	β	t
Gender	0.18**	4.17	0.19**	4.42	0.16**	4.14
Age	0.03	1.77	0.10**	6.69	0.06**	4.71
Perceived burdensomeness	0.32**	15.67	0.32**	15.69	0.18**	9.20
NSSI					0.29**	13.69
Digital self-harm					0.12**	5.50
Psychological capital					−0.14**	−7.23
Psychological capital ×NSSI					−0.03	−1.75
Psychological capital ×Digital self-harm					−0.08**	−5.54
*R^2^*	0.11		0.13		0.40	
*F*	89.56**		107.01**		179.01**	

**p < 0.01; NSSI = non-suicidal self- injury.

**Figure 3:**
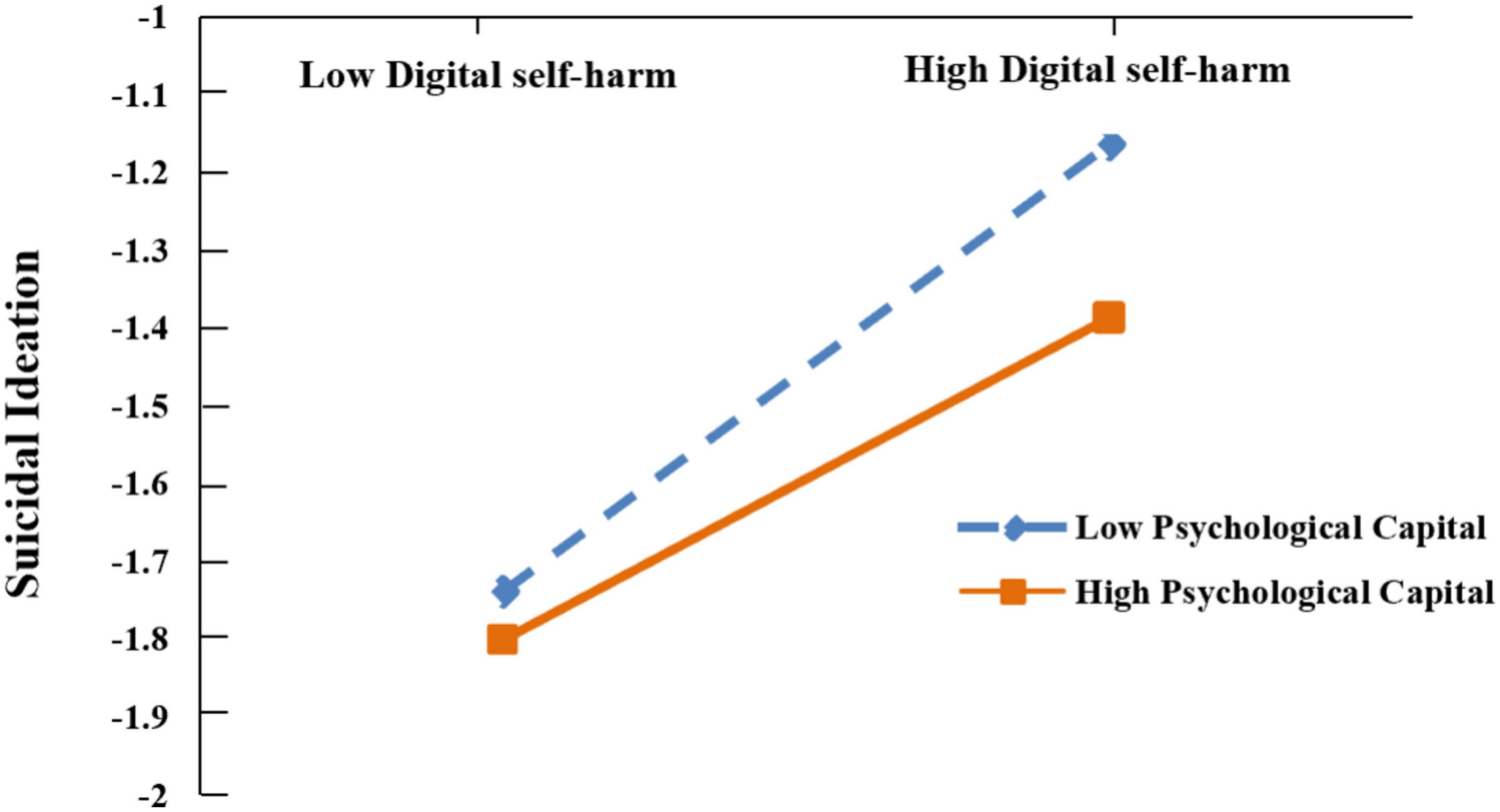
The moderating effect of psychological capital on the association between digital self-harm and suicidal ideation.

In Figure 3, simple slope analyses further showed that the effect of digital self-harm on suicidal ideation was significant at low levels of psychological capital (*β* = 0.19, *SE* = 0.02, *p* < 0.001), but not at high levels of psychological capital (*β* = 0.04, *SE* = 0.03, *p* = 0.19). Furthermore, for adolescents with low psychological capital, the indirect effect of perceived burdensomeness on suicidal ideation via digital self-harm was significant (*β* = 0.06, *SE* = 0.01, 95% CI [0.04, 0.08]), but this indirect effect was not significant among adolescents with high psychological capital (*β* = 0.01, *SE* = 0.01, 95% CI [−0.01, 0.04]).

## 4 Discussion

Although prior research has consistently identified perceived burdensomeness as one of the most stable and powerful psychological predictors of suicidal ideation (Van Orden et al., 2010; Chu et al., 2016), less is known about how it translates into suicidal ideation through specific behavioral mechanisms, and which factors can interrupt this risk pathway. To address this gap, the present study tested a moderated parallel mediation model. We examined the dual mediating roles of non-suicidal self-injury (NSSI) and digital self-harm in the association between perceived burdensomeness and suicidal ideation, as well as the moderating role of psychological capital. Our findings highlight the central role of self-harm behaviors in the development of suicide risk and reveal a context-specific buffering effect of psychological capital in digital environments.

### 4.1 Dual behavioral pathways linking perceived burdensomeness and suicidal ideation

The present study found that perceived burdensomeness not only directly predicted suicidal ideation but also indirectly predicted suicidal ideation through both NSSI and digital self-harm. Both behaviors served as partial mediators. These findings provide further support for the Interpersonal-Psychological Theory of Suicide (IPTS; Joiner, 2005) and align with the ideation-to-action framework, which conceptualizes self-harm as a key amplifier of suicide risk (Klonsky and May, 2015).

Regarding the NSSI pathway, our findings are consistent with extensive evidence showing that beliefs about being “a burden on others” often co-occur with intense self-disgust and shame, which are well-established psychological precursors of NSSI (Glassman et al., 2007; Hamza et al., 2012; Perez Rodríguez et al., 2025). Prior research indicates that NSSI serves emotion-regulatory functions and shows a robust association with suicidal ideation (Andover and Gibb, 2010; Guan et al., 2012; Hamza et al., 2012). By embedding this pathway within the IPTS framework, the present study further suggests that perceived burdensomeness may increase suicidal ideation by promoting engagement in NSSI among adolescents.

Importantly, we also found that perceived burdensomeness indirectly predicted suicidal ideation through digital self-harm. This finding extends previous research that has focused primarily on offline self-injury. Digital self-harm often involves anonymously posting self-directed negative content online and appears to serve functions similar to physical self-injury, including emotional expression and social reinforcement (Hooley and Franklin, 2018; Patchin and Hinduja, 2017). Recent studies further show that adolescents who engage in digital self-harm report higher levels of suicidal ideation and suicide attempts (Patchin and Hinduja, 2024; Wang et al., 2024). The present study is among the first to situate digital self-harm explicitly within the pathway of “perceived burdensomeness → self-harm → suicidal ideation.” These findings suggest that digital self-harm is not a peripheral phenomenon but a meaningful component of the suicide risk trajectory.

Notably, the direct effect of perceived burdensomeness on suicidal ideation was also significant, suggesting that behavioral pathways alone do not fully explain this relationship. This finding may be attributed to the intense feelings of “hopelessness” or “entrapment” triggered by perceived burdensomeness (O’Connor and Kirtley, 2018), through which severe psychological distress can directly translate into suicidal ideation without the mediating role of self-injurious behaviors. Such internal distress is likely intensified in the Chinese collectivistic context, where a strong emphasis on “shame culture” and the concept of “face” (mianzi) can exacerbate the detrimental impact of perceived burdensomeness (Chen et al., 2024). Specifically, when individuals perceive themselves as failing to meet familial expectations or the principles of “filial piety,” their sense of social responsibility may turn into profound self-devaluation, thereby accelerating the emergence of suicidal ideation (Ying et al., 2025).

### 4.2 The moderating role of psychological capital

This study further found that psychological capital significantly moderated the relationship between digital self-harm and suicidal ideation, but did not significantly moderate the relationship between NSSI and suicidal ideation. This result not only supports the fundamental view of psychological capital as a protective factor against suicide (Liu et al., 2023), but also reveals its path-specificity.

Specifically, digital self-harm significantly predicted suicidal ideation only among adolescents with low psychological capital, while this association became non-significant among those with high psychological capital. This selective buffering effect may reflect the socio-cognitive nature of digital self-harm. Compared with NSSI, digital self-harm relies more heavily on social evaluation, interpersonal feedback, and online self-presentation processes (Erreygers et al., 2020). Key components of psychological capital act as a “psychological shield”: self-efficacy reduces perceived social failure; hope and optimism enable positive reappraisal of negative feedback; and resilience facilitates rapid recovery from online pressure. Consequently, adolescents with high psychological capital may be more likely to cognitively reappraise negative online feedback and maintain hope, optimism, and self-worth (Liu et al., 2023; Shan et al., 2021). These capacities may prevent digital self-harm from escalating into suicidal ideation. In contrast, NSSI possesses stronger “physiological and automatic” attributes. It typically involves immediate physical pain and is driven by intense sensory stimuli, functioning as a highly automated emotion regulation reflex (Nock, 2010). Once NSSI evolves into a habitual coping mechanism, higher-order cognitive resources—such as psychological capital (e.g., hope and optimism)—may struggle to instantaneously interrupt the reflexive logic that progresses from NSSI to suicidal ideation.

### 4.3 Theoretical and practical implications

Theoretically, this study integrates IPTS with research on digital self-harm and responds to recent calls to recognize suicide risk as extending into digital ecosystems (Erreygers et al., 2020; Hinduja and Patchin, 2019). By distinguishing between NSSI and digital self-harm, the present findings demonstrate that protective factors do not function as universal buffers. Instead, they may exert differential effects across specific risk pathways.

Practically, these findings underscore the importance of interventions that directly target perceived burdensomeness, such as cognitive restructuring of the core belief “I am a burden” (Van Orden et al., 2010). The results also suggest that digital self-harm should be incorporated into school-based mental health screening and suicide risk assessments. This may be particularly critical for adolescents with low psychological capital, for whom digital self-harm may signal rapid escalation of suicide risk. Finally, the findings support psychological capital enhancement as a promising intervention target. Prior studies indicate that psychological capital interventions can increase adolescents’ hope and resilience and reduce suicide risk (Liu et al., 2023).

### 4.4 Limitations

Several limitations should be noted. First, this study relied on self-report measures, which may be influenced by social desirability and recall biases. Future research should incorporate multi-informant reports, interviews, and behavioral indicators. Second, the cross-sectional design precludes definitive conclusions regarding temporal precedence and causality, which inevitably weakens the robustness of the moderated mediation claims. Future research should implement planned longitudinal follow-ups or cross-lagged panel designs to establish the developmental sequencing of these variables. Specifically, tracking these constructs over multiple waves would allow for a more rigorous test of the “perceived burdensomeness → self-harm → suicidal ideation” pathway and help rule out potential reciprocal relationships among them. Third, while the single-item assessments for digital self-harm and suicidal ideation were selected to minimize participant burden in this large-scale survey and have been utilized in prior established literature (e.g., Lemke et al., 2025; Zhao et al., 2026), they may not capture the full multidimensionality of these constructs as effectively as multi-item instruments. Future studies should employ comprehensive validated scales—such as the Beck Scale for Suicide Ideation (SSI)—to report more robust psychometric properties and test–retest reliability. Fourth, the participants in this study were recruited from a single university, leading to high sample homogeneity. Future research should recruit cross-cultural and multi-sample (e.g., clinical samples) to enhance the generalizability of the findings. Additionally, given that psychological capital did not significantly moderate the NSSI pathway, future studies should explore other potential psychological factors, such as emotion regulation capacity, to more comprehensively elucidate the protective mechanisms against suicide risk.

## Data Availability

The raw data supporting the conclusions of this article will be made available by the authors, without undue reservation.
